# Adolescent Health Indicators: Violence Measures Are Critical to Include

**DOI:** 10.1016/j.jadohealth.2024.02.009

**Published:** 2024-06

**Authors:** Stephanie Burrows, Berit Kieselbach

**Affiliations:** Social Determinants of Health Department, World Health Organization, Geneva, Switzerland

Interpersonal violence is an important cause of mortality and morbidity among adolescents. It includes physical, sexual, and emotional violence and/or neglect and can be perpetrated by family members, peers, or community members, or in the context of gang activities [1]. Homicide is among the top five causes of death in adolescents, with boys comprising over 80% of victims and perpetrators [2]. Data from 2013 to 2017 show that approximately one in three adolescents aged 11–15 years were bullied in the past month, and one in three aged 13–15 years were involved in physical fights in the past year [3]. Bullying affects boys and girls in equal numbers; boys, however, tend to be more involved in physical violence and girls more in emotional violence. World Health Organization 2019 estimates show that one in four girls aged 15–19 years have experienced physical and/or sexual violence by their intimate partners [4].

Violence can cause severe immediate and long-term health and social consequences. Almost 54,000 adolescents are estimated to die each year as a result of interpersonal violence [5]. Many more suffer severe injuries and disability. Exposure to violence also results in long-term consequences, affecting neurological and mental health, cardiovascular, immune, and other biological systems. High-risk health behaviors such as unsafe sex, harmful alcohol and drug use, and smoking are more frequent among victims of violence and, in turn, contribute to lifelong ill health and premature mortality. Violence can also affect cognitive development and academic performance, with higher school drop-out among victims, and increases the likelihood of perpetrating violence later in life.

To prevent violence against adolescents and develop effective interventions, better data on the burden of adolescent violence are needed, especially from low-income and lower-middle-income countries, where the burden is highest [6]. However, very few adolescents disclose abuse and less than 12% receive help from official services [7]. Prevalence estimates from administrative data used by health or justice systems reflect only those who use these services and therefore vastly underestimate the true magnitude of violence against adolescents. Nationally representative survey data are therefore critical in providing a more realistic picture of the burden, yet few countries have nationally representative data for all types of violence, and even fewer collect data at regular intervals. For example, the Violence Against Children and Youth Survey—a comprehensive survey covering physical, emotional, and sexual violence against children and youth, risk and protective factors, and the impact of violence and perpetration—has been or is being carried out in only 23 countries and has been repeated in only three countries so far. Across different surveys and countries, comparisons are hampered by variations in definitions of violence, recall periods, and age groups. For the most part, surveys collect data on some forms of violence only and rarely measure certain forms of violence, like sexual violence against boys. Very few surveys ask about who perpetrated the violence, information which is essential to develop effective interventions.

The adolescent health indicators recommended by the Global Action for Measurement of Adolescent Health (GAMA) Advisory Group cover several determinants of health and high-risk health behaviors that are highly relevant to understanding and preventing violence. Measuring violence as part of a broader range of adolescent health indicators makes it possible to assess and monitor changes in several interconnected indicators, thereby providing a richer understanding. The violence-related indicators cover bullying, physical violence, and sexual violence (see Table 1). All violence-related indicators recommend disaggregation by sex and age, and in the context of bullying also by type of bullying. Sex and age disaggregated data will help countries to understand what interventions work best for males and females and different adolescent age groups [8].

**Table 1:** Recommended violence-related indicators

Indicator	Violence type covered	Age group covered	Age group providing data	Recall period	Recommended disaggregation
Bullying	Bullying (in-person, digital/cyber)	10–19 years (10–14, 15–19 years)	10–19 years	Past year	Age, sex, type of bullying
Physical violence experience	Physical violence	10–19 years (10–14, 15–19 years)	10–19 years	Past year	Age, sex
Contact sexual violence experience	Contact sexual violence	10–19 years (10–14, 15–19 years)	10–19 years	Past year	Age, sex
Sexual violence experience by age 18 (additional indicator)	Sexual violence	0–18 years (<10, 10–14, 15–18 years)	18–29 years	Lifetime	Age, sex

The GAMA-recommended indicators cover important types of violence affecting adolescents. Regular collection of these data at the country level would be a huge benefit for the field of violence prevention. While the issues of measurability, availability of data, and parsimony are important considerations, in future iterations ideally the indicators would also include other forms of sexual violence that do not involve physical contact such as in-person verbal sexual harassment, being forced to undress, exposure, or online sexual abuse; and emotional violence such as repeated belittling, degrading, shaming, or ridiculing that is perpetrated by adults (emotional violence by peers is included under bullying). These forms of violence often occur concurrently with physical forms. Furthermore, while collecting data on perpetration can present ethical challenges, including the duty to report in some countries, knowing who the perpetrator is provides critical information for intervention development. If appropriate and feasible, countries would ideally collect this information.

While some surveys partially collect data for these indicators, GAMA proposes to include younger age groups, who carry a large burden of the violence and have so far been neglected. Concerns about asking young adolescents sensitive questions about violence have been raised but reviews show that participant distress is infrequent and transitory if safeguarding protocols are in place [9,10]. Indeed, collecting information from adolescents requires strict ethical procedures—in addition to consent and confidentiality, there should be clearly defined protocols and strategies for implementation for limiting distress and providing support and resources where needed. It will be critical for entities adopting the measurement of the adolescent health priority indicators to have such safeguarding in place.

The GAMA-recommended indicators provide a coherent selection of critical indicators to measure health behaviors and determinants, outcomes, and policy and program implementation of relevance to adolescents. In the ongoing refinement of these indicators, we would propose the inclusion of variables such as access to firearms and weapon carrying, in addition to those on substance and alcohol use. The violence-related indicators form an essential part of this package as adolescent health cannot be achieved without measuring and addressing violence. There are good arguments for including the younger age group of adolescents and the disaggregation proposed by GAMA but data collection should not be blocked in settings where this is not possible. Several existing nationally representative surveys, including those focused specifically on violence like the Violence Against Children and Youth Survey or those covering broader health issues such as the Multiple Indicator Cluster Surveys, the Demographic and Health Surveys, the Global School-based Student Health Surveys, and the Health Behaviour in School-aged Children Surveys provide violence-related data very similar to the GAMA-recommended indicators. These data are critical for assessing the burden of violence, developing and implementing appropriately targeted policies and programs, and monitoring progress over time and countries should be encouraged to collect it on a regular basis.
